# ST-segment elevation myocardial infarction as the first clue to membranous nephropathy: A case report

**DOI:** 10.1097/MD.0000000000047502

**Published:** 2026-02-06

**Authors:** Dongmei Wang, Hua Deng, Miao He, Tianyu He

**Affiliations:** a Department of Cardiology, Mianyang Fulin Hospital, Mianyang, China; b Sichuan College of Traditional Chinese Medicine, Mianyang, China.

**Keywords:** acute myocardial infarction, cardiorenal syndrome, coronary artery, membranous nephropathy, ST elevation

## Abstract

**Rationale:**

Acute ST-segment elevation myocardial infarction (STEMI) typically results from abrupt coronary occlusion, most often caused by rupture of an atherosclerotic plaque. Membranous nephropathy (MN) typically presents with nephrotic syndrome, characterized by proteinuria. STEMI as the initial manifestation of MN is exceptionally rare. This case underscores the importance of considering systemic hypercoagulable states when evaluating atypical myocardial infarction in young adults.

**Patient concerns:**

A 35-year-old man with no prior history of renal impairment, cardiovascular disease, or risk factors such as hypertension or diabetes, presented with acute chest pain and was diagnosed with STEMI. Subsequent evaluation revealed MN with nephrotic-range proteinuria. 110 days after discharge, he experienced a recurrent STEMI.

**Diagnoses:**

The 12-lead electrocardiogram demonstrated ST-segment elevation in leads II, III, and augmented vector foot, consistent with inferior-wall myocardial infarction. Emergency coronary angiography showed acute occlusion of the RCA. In the setting of nephrotic-range proteinuria and hypoalbuminemia, the infarct mechanism was most consistent with coronary thrombosis secondary to a nephrotic syndrome-associated hypercoagulable state.

**Interventions:**

During coronary angiography, aspiration thrombectomy was performed, and systemic anticoagulation and guideline-directed secondary prevention were initiated.

**Outcomes:**

Coronary flow was restored to thrombolysis in myocardial infarction grade 3, and the patient became hemodynamically stable. He was transferred to the nephrology service for targeted management of MN. One hundred ten days later, he represented with ST-segment elevation. Coronary angiography demonstrated thrombolysis in myocardial infarction grade 3 flow with only mild mid-to-distal RCA stenosis, raising suspicion of a transient coronary embolic event.

**Lessons:**

Although rare, STEMI may be the initial manifestation of MN. In young patients with myocardial infarction who exhibit clinical features of nephrotic syndrome, a hypercoagulable state should be considered. Early recognition, timely initiation of antithrombotic therapy, and multidisciplinary follow-up are essential to reduce the risk of recurrence.

## 1. Introduction

Acute ST-segment elevation myocardial infarction (STEMI) most commonly results from acute occlusion of a coronary artery caused by atherosclerotic plaque rupture. Acute myocardial infarction (AMI) occurs when coronary occlusion is sustained long enough to cause irreversible myocardial injury. This process is typically triggered by an abrupt reduction or interruption of coronary blood flow, leading to extensive and prolonged ischemic injury in the affected myocardial territory.^[1]^ Less commonly, STEMI results from coronary ostial obstruction or coronary embolism due to other rare etiologies. In clinical practice, identifying the precipitating cause of coronary ischemia is critical to delivering timely, appropriate reperfusion therapy and limiting myocardial necrosis.

Membranous nephropathy (MN) is a leading cause of nephrotic syndrome in adults. The nephrotic state—characterized by heavy proteinuria, hypoalbuminemia, hyperlipidemia, and edema—is associated with a prothrombotic (hypercoagulable) state. Venous thromboembolism is well recognized; arterial thrombosis, although less frequent, can involve the coronary, cerebral, and peripheral arterial beds.^[2]^

We describe a young adult in whom an inferior-wall STEMI provided the first clinical clue to MN. We outline the diagnostic reasoning, differential diagnoses, therapeutic decisions, and follow-up considerations. For clarity and reproducibility, we provide detailed timelines, imaging, and laboratory data.

## 2. Case report

**Figure 1.**
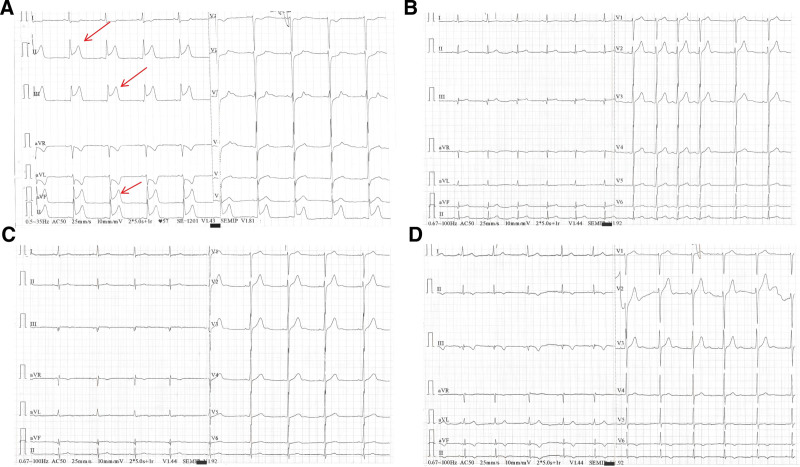
The ECG at the time of the first episode. (A) Preoperative ECG showed revealed significant ST-segment elevation in leads II, III, and aVF. (B) The ECG on the postoperative day was normal. (C) The ECG was normal on the first postoperative day. (D) The ECG was normal on the second postoperative day. aVF = augmented vector foot, ECG = electrocardiogram.

**Figure 2.**
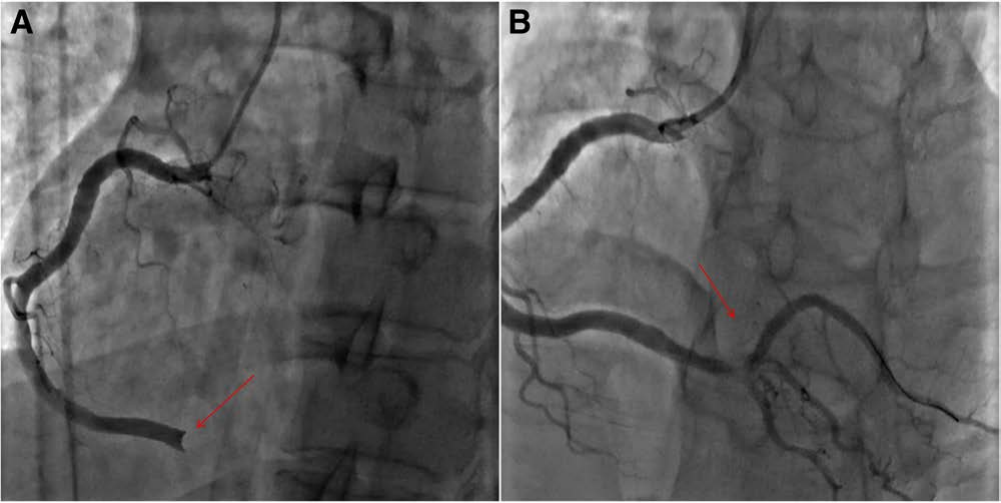
Comparison before and after emergency coronary angiography. (A) Emergency coronary angiography revealed coronary occlusion. (B) The distal blood flow returned to normal after emergency coronary thrombus aspiration.

On February 22, 2024, a 35-year-old man developed sudden chest pain at rest, characterized by persistent precordial pain radiating to the shoulder and back, accompanied by chest tightness and profuse sweating. The pain did not subside with rest, prompting immediate presentation to the emergency department. An emergency electrocardiogram (ECG) showed ST-segment elevation (Fig. 1A). Coronary angiography demonstrated: no significant stenosis of the left main coronary artery; no significant stenosis of the proximal left anterior descending or left circumflex arteries, with thrombolysis in myocardial infarction (TIMI) grade 3 flow; and acute occlusion of segment 3 of the right coronary artery (RCA) with visible thrombus and TIMI 0 flow (Fig. 2A). He was diagnosed with coronary artery disease and an acute inferior-wall STEMI.

After informed consent, percutaneous coronary intervention of the RCA was performed. Aspiration thrombectomy removed a large thrombus and restored TIMI grade 3 flow (Fig. 2B). The patient was then transferred to the coronary care unit. On admission, vital signs were stable: temperature 36.5°C; pulse 85 beats/min; respiratory rate 19 breaths/min; and blood pressure 113/75 mm Hg. The patient’s body mass index was 25.68 (weight: 86 kg, height: 183 cm). He had no prior history of hypertension, diabetes, renal disease, or cardiovascular disease, and had no smoking history. No edema was present. He was alert and in no acute distress. Cardiovascular, respiratory, abdominal, and neurological examinations were unremarkable. Laboratory tests revealed d-dimer of 0.29 mg/L, HR-7 prothrombin time of 10.4 seconds, HR-7 prothrombin time ratio of 0.89, fibrinogen activity of 111.7, HR-7 activated partial thromboplastin time of 240.6 seconds↑, HR-7 fibrinogen of 2.91 g/L, and thrombin time > 360.00 seconds↑. These results suggest a hypercoagulable state. Follow-up ECG showed partial resolution of the ST-segment elevation (Fig. 1B–D).

Post-percutaneous coronary intervention, guideline-directed secondary prevention was initiated: rivaroxaban 15 mg daily, enteric-coated aspirin 100 mg daily, clopidogrel 75 mg daily (dual antiplatelet therapy); atorvastatin 20 mg at bedtime; metoprolol succinate 47.5 mg daily; and extended-release isosorbide mononitrate 30 mg daily. A transthoracic echocardiogram showed a left ventricular ejection fraction of 66% and fractional shortening of 36%. There were no structural cardiac abnormalities, and both systolic and diastolic functions were normal. Chest radiography showed no cardiopulmonary abnormalities. Complete blood count was normal, but serum total protein was low at 36.4 g/L (reference range 60–83 g/L) and albumin at 24.0 g/L (35–55 g/L). The lipid profile showed elevated total cholesterol (6.04 mmol/L; ≤5.20 mmol/L), triglycerides (4.28 mmol/L; ≤2.30 mmol/L), and low-density lipoprotein cholesterol (3.65 mmol/L; ≤3.36 mmol/L). Urinalysis showed 3 + proteinuria, and 24-hour urinary protein excretion was 6.08 g. On the basis of these findings, nephrotic syndrome was diagnosed, and the patient was transferred to the nephrology department for treatment. Renal biopsy confirmed membranous nephropathy. His condition improved after treatment, and he was discharged. After discharge, the patient was prescribed long-term oral medications: enteric-coated aspirin 100 mg daily, clopidogrel 75 mg daily, atorvastatin 20 mg nightly, and sacubitril–valsartan 100 mg twice daily to reduce urinary protein excretion. The patient was also instructed to follow-up regularly at outpatient clinics, where medication adjustments and monitoring were continued.

**Figure 3.**
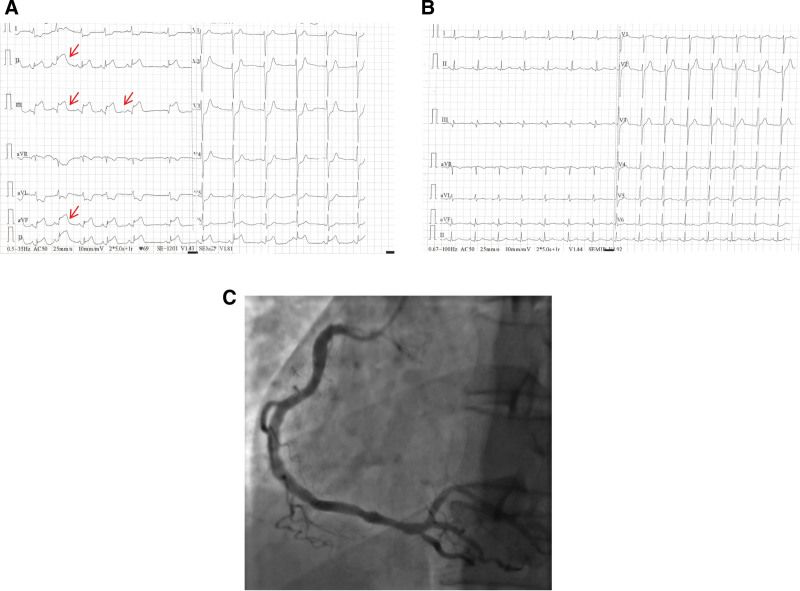
The ECG and coronary angiography at the time of the second episode in June 2024. (A) Preoperative ECG showed revealed significant ST-segment elevation in leads II, III, and aVF. (B) The ECG returned to normal after coronary angiography. (C) Emergency coronary angiography. aVF = augmented vector foot, ECG = electrocardiogram.

On June 11, 2024, the patient again presented with chest pain. ECG showed sinus rhythm with ST-segment elevation in leads II, III, and augmented vector foot (Fig. 3A), confirming an acute inferior-wall STEMI. Immediate anticoagulation and antiplatelet therapy were started. Coronary angiography demonstrated no significant stenosis of the left main coronary artery; no significant stenosis of the proximal left anterior descending or left circumflex arteries, with TIMI grade 3 flow; and ~20% stenosis in the mid and distal RCA, with TIMI grade 3 flow (Fig. 3C). A coronary embolism, likely self-limited, was diagnosed. The patient was hospitalized, with continued anticoagulation and active nephrotic syndrome treatment. He was discharged after clinical improvement (Fig. 3B).

The patient is clinically stable with regular follow-up and no further myocardial infarctions. To support secondary prevention after STEMI and manage membranous nephropathy-related hypercoagulability, the following follow-up plan was implemented: a review at 2 weeks post-discharge (cardiology + nephrology), then monthly for the first 3 months, every 6 to 8 weeks in months 4 to 6, every 2 to 3 months in months 7 to 12, and annually thereafter if stable. Each visit includes symptom and vital sign assessment, functional class evaluation, and a 12-lead ECG for the first 3 months. Echocardiography is performed post-discharge and at 3 to 6 months if needed. Laboratory tests include a lipid panel at baseline and 6 to 8 weeks after statin initiation, then every 3 to 6 months, with liver enzymes monitored during statin therapy. Nephrology monitoring includes 24-hour urine protein (or UPCR) every 4 to 8 weeks until sub-nephrotic, then every 3 months, along with albumin, creatinine/estimated glomerular filtration rate, and electrolytes at the same intervals. Antithrombotic therapy is adjusted as albumin rises and proteinuria decreases. Red-flag education and coordinated cardiology–nephrology care are emphasized. A detailed timeline is shown in Table 1.

**Table 1:** Timeline of clinical events.

Date/time	Clinical events	Diagnostic assessments	Therapeutic interventions	Outcomes
February 2024 (index)	Acute chest pain → ED	ECG: inferior STEMI; CAG: RCA seg.3 occlusion (TIMI 0)	Primary PCI + thrombectomy → TIMI 3; CCU; DAPT + rivaroxaban; atorvastatin; metoprolol; ISMN	Stable post-PCI
Post-procedure day 0	Routine post-op assessment	Echo: LVEF 66%, FS 36%; no structural dysfunction	–	No complications
Day 0–2	Telemetry	Serial ECG: ST resolution by day 2	–	Uneventful
Inpatient labs (February 2024)	Edema/proteinuria work-up	Albumin 24 g/L; TP 36.4 g/L; TC 6.04; TG 4.28; LDL-C 3.65; urine protein 3+; 24-h 6.08 g	Dx: nephrotic syndrome	Further work-up
February 2024 (later)	Renal biopsy	Membranous nephropathy	Nephrology therapy; discharge	Proteinuria managed
June 2024 (recurrence)	Recurrent chest pain	ECG: inferior STEMI	Immediate antithrombotics	Proceed to CAG
June 2024 (during stay)	Coronary reassessment	CAG: LM/LAD/LCX no sig lesions; RCA mid-distal ~20% (TIMI 3)	Transient embolism suspected; continue AC; intensify nephrotic syndrome Rx	Symptoms resolved; discharge
Follow-up (present)	Outpatient follow-up	–	Maintenance secondary prevention + nephropathy management	Stable; no recurrent MI

ECG = electrocardiogram, CCU = coronary care unit, LAD = left anterior descending, MI = myocardial infarction, PCI = percutaneous coronary intervention, STEMI = ST-segment elevation myocardial infarction, TIMI = thrombolysis in myocardial infarction.

## 3. Discussion

This case illustrates an unusual presentation of AMI as the first manifestation of MN in a young patient with no significant cardiovascular risk factors. The patient’s presentation with AMI highlights the need for a thorough investigation of systemic causes in the absence of coronary artery disease. AMI should always be considered in the differential diagnosis of unexplained chest pain.

AMI is a leading cause of morbidity and mortality worldwide.^[3]^ Most AMI arises from coronary atherosclerotic disease. However, some cases occur in the absence of obstructive coronary artery disease.^[4]^ Other mechanisms include spontaneous coronary artery dissection (SCAD), coronary spasm, hypercoagulability, coronary embolism, coronary vasculitis, myocardial oxygen supply–demand mismatch, extrinsic compression, allergic reactions, and noncardiac causes.^[5–8]^ In the absence of atherosclerosis, acute coronary embolism should prompt an evaluation for underlying systemic causes.

Harish et al^[9]^ reported STEMI as the initial presentation of AMI in a patient with an underlying hematological disorder. This case, however, is unique in that AMI was the first manifestation of MN.

Vasculitis and SCAD are important differential diagnoses in patients presenting with acute coronary events. Vasculitis can lead to coronary inflammation, wnich may cause subsequent arterial thrombosis or aneurysm formation, potentially mimicking AMI.^[10]^ In SCAD, there is spontaneous tearing of the coronary artery wall, often without prior atherosclerotic disease. These conditions may present with similar acute chest pain and ST-segment elevation, requiring differentiation through coronary angiography or advanced imaging techniques such as intravascular ultrasound or optical coherence tomography.^[11]^ Addressing these differential diagnoses is crucial for accurate diagnosis and appropriate treatment. Furthermore, it is essential to consider the role of hypercoagulability and the increased risk of thrombosis in patients with nephrotic syndrome when determining anticoagulation and antiplatelet therapy strategies.

MN is more common in middle-aged and older adults and is more prevalent in males. The typical age at onset is 50 to 60 years, although recent series suggest a trend toward younger patients.^[12]^ Early MN often presents with few symptoms; approximately 70%–80% of patients manifest nephrotic syndrome. About 30% have microscopic hematuria, whereas gross hematuria is rare.^[13]^ Venous thromboembolism is common in MN, whereas arterial events are less frequent. Reported thrombotic events in nephrotic syndrome include thrombosis of the lower extremity artery,^[14]^ aortic valve,^[15]^ coronary artery,^[16]^ and cerebrovasculature,^[17]^ as well as acute arterial embolism.^[18]^ Acute myocardial infarction has also been reported in pediatric patients with nephrotic syndrome.^[19]^ However, to our knowledge, acute STEMI as an initial manifestation of MN or nephrotic syndrome has not been previously reported.

Nephrotic syndrome is associated with hemoconcentration (reduced effective circulating volume) and hyperlipidemia, which increase blood viscosity. Urinary loss of anticoagulant proteins triggers compensatory hepatic synthesis, producing imbalance across the coagulation, anticoagulation, and fibrinolytic systems. These changes create a prothrombotic state that predisposes to arterial and venous thrombosis, including myocardial infarction. Guidelines on anticoagulation therapy in nephrotic syndrome emphasize the importance of balancing the risks of bleeding and thrombosis. In such patients, low-molecular-weight heparin or direct oral anticoagulants can be used to prevent thrombotic events, with close monitoring of coagulation profiles.^[20,21]^ Early recognition and treatment of the underlying disorder can substantially improve prognosis. In hypercoagulable patients, management should prioritize treatment of the primary disease and close monitoring to reduce the risk of recurrent myocardial infarction.

In this case, the patient initially presented with coronary thrombosis and, more than 110 days later, experienced recurrent coronary embolism—an exceedingly rare pattern. In nephrotic syndrome, although vigilance often centers on venous thrombosis, the possibility of arterial embolism should not be overlooked. Such cases may warrant intensified anticoagulation and antiplatelet therapy, alongside aggressive treatment of the underlying disease to prevent recurrent arterial embolism. In addition to membranous nephropathy, other conditions that contribute to a hypercoagulable state, such as autoimmune disorders, malignancies, and inherited thrombophilias, should be considered in similar cases. These conditions may exacerbate the risk of thrombosis and complicate treatment strategies.

This case has several limitations, including the inability to establish causality due to the observational nature of the case report. The findings may not be generalizable to other populations, and there is a risk of selection bias as the diagnosis was guided by the patient’s presentation. Further confirmatory testing, such as genetic screening for thrombophilic disorders, could have helped to elucidate the underlying cause of the hypercoagulable state.

## 4. Conclusion

This case also underscores the need for timely diagnosis, appropriate antithrombotic therapy, and a multidisciplinary approach to manage both the primary disease and the cardiovascular complications. Further research is required to better understand the mechanisms linking nephrotic syndrome and myocardial infarction, as well as to explore the best strategies for secondary prevention in similar cases.

## Acknowledgments

We would like to express our gratitude to the patient for granting permission to use their clinical data in this paper and for the publication of this research.

## Author contributions

**Methodology:** Hua Deng.

**Writing – original draft:** Dongmei Wang.

**Writing – review & editing:** Dongmei Wang, Hua Deng, Miao He, Tianyu He.
